# Positive Selection and Increased Antiviral Activity Associated with the PARP-Containing Isoform of Human Zinc-Finger Antiviral Protein

**DOI:** 10.1371/journal.pgen.0040021

**Published:** 2008-01-25

**Authors:** Julie A Kerns, Michael Emerman, Harmit S Malik

**Affiliations:** 1 Division of Basic Sciences, Fred Hutchinson Cancer Research Center, Seattle, Washington, United States of America; 2 Division of Human Biology, Fred Hutchinson Cancer Research Center, Seattle, Washington, United States of America; University of Oxford, United Kingdom

## Abstract

Intrinsic immunity relies on specific recognition of viral epitopes to mount a cell-autonomous defense against viral infections. Viral recognition determinants in intrinsic immunity genes are expected to evolve rapidly as host genes adapt to changing viruses, resulting in a signature of adaptive evolution. *Zinc-finger antiviral protein (ZAP)* from rats was discovered to be an intrinsic immunity gene that can restrict murine leukemia virus, and certain alphaviruses and filoviruses. Here, we used an approach combining molecular evolution and cellular infectivity assays to address whether *ZAP* also acts as a restriction factor in primates, and to pinpoint which protein domains may directly interact with the virus. We find that *ZAP* has evolved under positive selection throughout primate evolution. Recurrent positive selection is only found in the poly(ADP-ribose) polymerase (PARP)–like domain present in a longer human *ZAP* isoform. This PARP-like domain was not present in the previously identified and tested rat *ZAP* gene. Using infectivity assays, we found that the longer isoform of ZAP that contains the PARP-like domain is a stronger suppressor of murine leukemia virus expression and Semliki forest virus infection. Our study thus finds that human *ZAP* encodes a potent antiviral activity against alphaviruses. The striking congruence between our evolutionary predictions and cellular infectivity assays strongly validates such a combined approach to study intrinsic immunity genes.

## Author Summary

Host–virus interactions are a classic example of genetic conflict in which both entities try to gain an evolutionary advantage over the other. This “back-and-forth” evolution is predicted to result in rapid changes of both host and viral proteins, which results in an evolutionary signature of positive selection, especially at the direct interaction interface. Recent studies have demonstrated that host proteins can target intracellular stages of the viral life cycle to potently inhibit viruses. Collectively, these host proteins are referred to as “intrinsic immunity” proteins. One such protein, zinc-finger antiviral protein (ZAP), was previously described from rats and shown to inhibit retroviruses and alphaviruses. We queried the primate orthologs of ZAP to ascertain both whether they have evolved under positive selection, and whether they have antiviral activity. We found that the signature of positive selection was restricted to a poly(ADP-ribose) polymerase–like domain in a longer isoform of primate ZAP. The longer human ZAP isoform has increased antiviral activity against both retroviruses and alphaviruses. Thus, positive selection correctly predicted the more potent antiviral isoform of this protein.

## Introduction

Recent discoveries have highlighted the role of intrinsic immunity genes in primate host defense against viral infections [1–3]. These genes are predicted to be locked in ancient, ongoing genetic conflicts with an ever-changing repertoire of viral infections [4–6]. Consistent with this prediction, the primate genes that encode for intrinsic immunity have been found to be evolving under positive selection, wherein they accumulate an excess number of non-synonymous substitutions (protein-altering, dN) compared to synonymous substitutions (no effect on protein, dS). This kind of selective pressure is seen in cases where innovation in protein sequence can result in a selective advantage and rapid fixation, as is the case for a host immunity gene where a single mutation might improve its ability to recognize and destroy a pathogen. In fact, genome-wide scans for positively selected genes in primates reveal that adaptively evolving genes fall primarily into three functional categories: immune defense, chemosensory perception, and reproduction, with the majority of these genes being involved in immunity [7].

Four known groups of intrinsic immunity genes have been preserved over a broad taxonomic range; *APOBEC3G* and other *APOBEC3* genes as well as *TRIM5* are conserved across many mammalian orders [8,9], while *Fv1* is conserved in many *Mus* species [10]. All three of these groups of these intrinsic immunity genes have been shown to evolve under positive selection [4–6,10]. A signature of positive selection has not only provided information about the antiviral activity and age of these “restriction” genes, but has also helped to identify protein domains at the direct interface of the host–virus interaction [4].

The fourth intrinsic immunity gene that is present over a broad taxonomic range is the *zinc-finger antiviral protein (ZAP). ZAP* from rat cells (referred to as *rZAP*) was identified due to its ability to significantly impair the replication of the mouse retrovirus, murine leukemia virus (MLV), through a mechanism that involves binding and degrading viral RNAs in the cytoplasm [11]. Viral RNA recognition by rZAP is mediated by the 4 CCCH zinc finger motifs, which have been shown to directly bind viral RNA with high specificity [12]. ZAP-dependent recruitment of an RNA processing exosome in the cytoplasm then leads to viral RNA degradation [13]. Subsequent studies have found that rZAP can inhibit both alphaviruses [14,15] and filoviruses [16] by inhibiting the translation of incoming viral RNA. We undertook a combined approach using an evolutionary analysis of *ZAP* orthologs in primates as well as functional tests of ZAP isoforms from humans.

The human ortholog of the *ZAP* gene encodes 2 protein isoforms that result from alternative splicing of a carboxy-terminal poly(ADP-ribose) polymerase (PARP)-like domain. This PARP-like domain is present in *ZAP(L)* and absent in *ZAP(S).* Two shorter rat *ZAP* isoforms have previously been described, one corresponding to human ZAP(S) and the other to just the first 254 amino acids of the N-terminus (rat NZAP), and neither contain the PARP-like domain[11]. Our evolutionary analysis of *ZAP* revealed strong evidence of positive selection throughout primate evolution. By examining the pattern of evolutionary change in this gene, we found, surprisingly, no evidence for positive selection in *ZAP*'s CCCH RNA-binding domain. This implies that rapid alterations of viral mRNA binding have not driven *ZAP* evolution. Instead, the signature of positive selection in primate *ZAP* is confined to the PARP-like domain, implicating this domain as an uncharacterized and evolutionarily important interface for ZAP–virus interactions. Using infectivity assays, we show that human ZAP is capable of restricting expression of the retrovirus, MLV, as well as infection by the alphavirus, Semliki Forest virus (SFV), in human cells. Moreover, we show that the presence of the PARP-like domain in the longer human ZAP isoform significantly enhances this restrictive capability against both viruses, making this the first demonstration of a PARP-like domain being implicated in an immunity role. Our results demonstrate that a combination of evolutionary and virological analyses can identify and validate specific protein domains involved in host–pathogen interactions, thereby uncovering previously unknown antiviral activity.

## Results

### Two Predominant *ZAP* Isoforms

**Figure 1:**
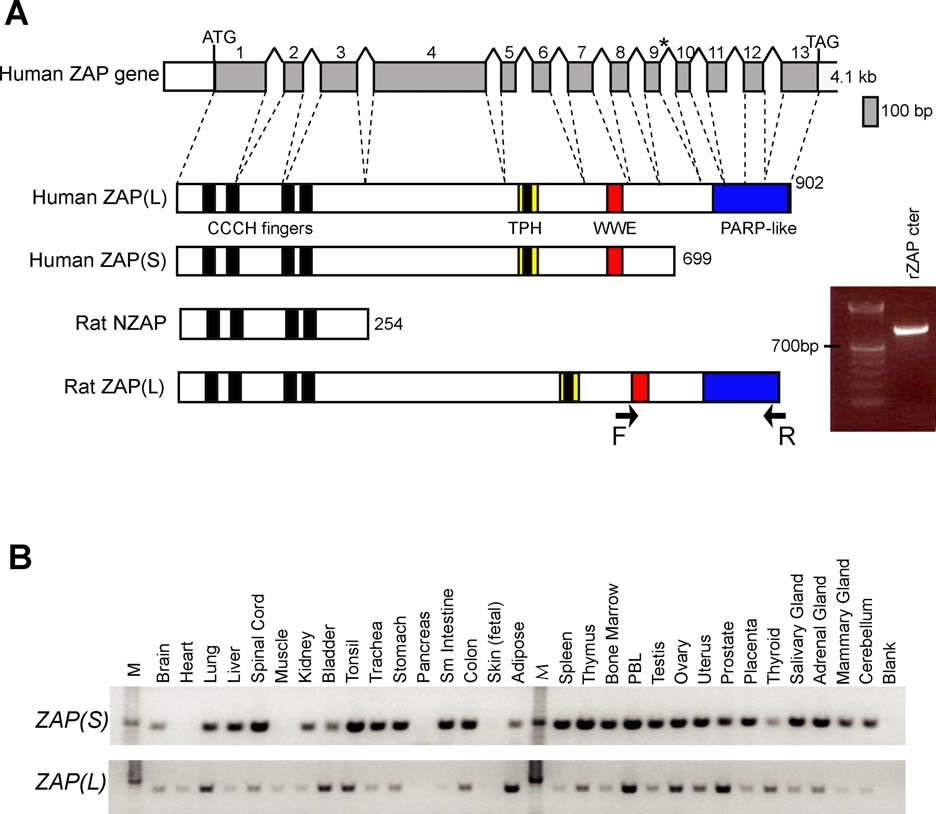
*ZAP(S)* and *ZAP(L)* Isoforms in Mammals (A) Schematic of the genomic structure of human *ZAP(L)* and the protein structure with previously defined domains (CCCH fingers, TPH, WWE, and PARP-like). The asterisk indicates the stop codon for an alternative transcript that has been reportedly isolated from multiple cDNA libraries. An additional transcript with an alternatively spliced exon (366 bp) inserted after exon 4 was also identified in several primates (not shown) but not in human, so our evolutionary analysis was limited to the *ZAP(L)* isoform shown. The rat NZAP (254 aa) protein structure is shown for comparison. A longer *ZAP(L)* isoform was also detected by RT-PCR from rat liver RNA using a forward primer in the WWE domain (exon 7) and a reverse primer in the PARP domain (exon 13) (see arrows). The resulting product was directly sequenced and corresponded to exons 7–13 of rat *ZAP* (gel inset). (B) Human ZAP isoforms are expressed in a wide range of tissues, including germline tissues and peripheral blood lymphocytes (PBLs). Results from PCR amplification from a human multiple tissue cDNA panel with primers specific to the human *ZAP(S)* and *(L)* isoforms (top and bottom panels, respectively). Lanes are labeled according to the template tissue (M, DNA standard marker; blank, no template).

The human *ZAP* gene comprises 13 exons spanning approximately 60 kb (Figure 1A). The two reported alternatively spliced isoforms of human *ZAP* code for proteins that either lack or contain a carboxy-terminal PARP-like domain (Refseq NM_020119.3 and NM_024625.3). We refer to the short and long isoforms as *ZAP(S)* and *ZAP(L),* respectively (Figure 1A). The *ZAP(L)* isoform encodes a protein with an N-terminal CCCH domain (four CCCH motifs), a TPH, or TiPARP Homology domain (conserved among ZAP paralogs and containing a fifth zinc finger motif), a WWE domain (predicted to mediate specific protein–protein interactions in ubiquitin and ADP–ribose conjugation proteins [17]) and a C-terminal PARP-like domain. The antiviral activity of *rZAP* was first discovered using a truncated rat ZAP protein (rat NZAP), which consisted only of the 4 CCCH motifs that mediate RNA binding [11] (Figure 1A). Subsequent analyses revealed that a longer *rZAP* protein also has antiviral ability. However, even this rat ZAP, presumed at the time to be “full length,” corresponds to the human *ZAP(S)* isoform and does not include the C-terminal PARP domain.

To gauge the tissue-specificity of the two human isoforms relative to each other, we used RT-PCR from a human tissue cDNA panel and primers specific to the *ZAP(S)* and *ZAP(L)* isoforms (Figure 1B). We found that *ZAP(S)* is expressed has a broader expression pattern compared to the *ZAP(L)* isoform. However, the *ZAP(L)* isoform is expressed in tissues where it may mediate an intrinsic immunity function, including germline tissues and peripheral blood lymphocytes (PBLs). Indeed, the tissue range of *ZAP(L)* is comparable to some of the *APOBEC3* genes that we have analyzed previously using the same cDNA panel [6].

Next, we wanted to address whether *ZAP(L)* is limited to the primate lineage. The Refseq entries for both mouse and rat ZAP proteins only refer to a protein equivalent of human *ZAP(S)*. On examining the genomic sequence context for both rat and mouse *ZAP* genes, we found that they both have a downstream set of exons that could encode a putative PARP domain that would be orthologous to that from primate *ZAP(L)*. In order to determine whether these exons are spliced onto the remainder of the *ZAP* gene, we performed RT-PCR using RNA from rat liver and primers designed to the WWE domain (shared between both *ZAP(S)* and *ZAP(L)* isoforms) and the PARP domain. Indeed, we found that the rat *ZAP* gene can encode a *ZAP(L)* isoform that includes a C-terminal PARP domain (Figure 1A). The reason this isoform appears to have been missed until now is because no ESTs corresponding to this PARP domain have been reported, suggesting that *ZAP(L)* may be more weakly expressed than *ZAP(S)* in rodents, as is the case in humans (Figure 1B).

In terms of overall architecture and paralogous genes, *ZAP* is most closely related to 3 other PARP-containing genes: *PARP11, PARP12,* and *TiPARP* [18] as well as *ZRP2*, which only consists of a CCCH domain (Figure S1). Putative orthologs for all five genes can be found in genome sequences from a variety of mammals, as well as chicken and fish, suggesting that this gene family is ancient [19] and that the PARP-containing ZAP isoform dates back to the origin of vertebrates.

### Positive Selection of Primate *ZAP*

**Figure 2:**
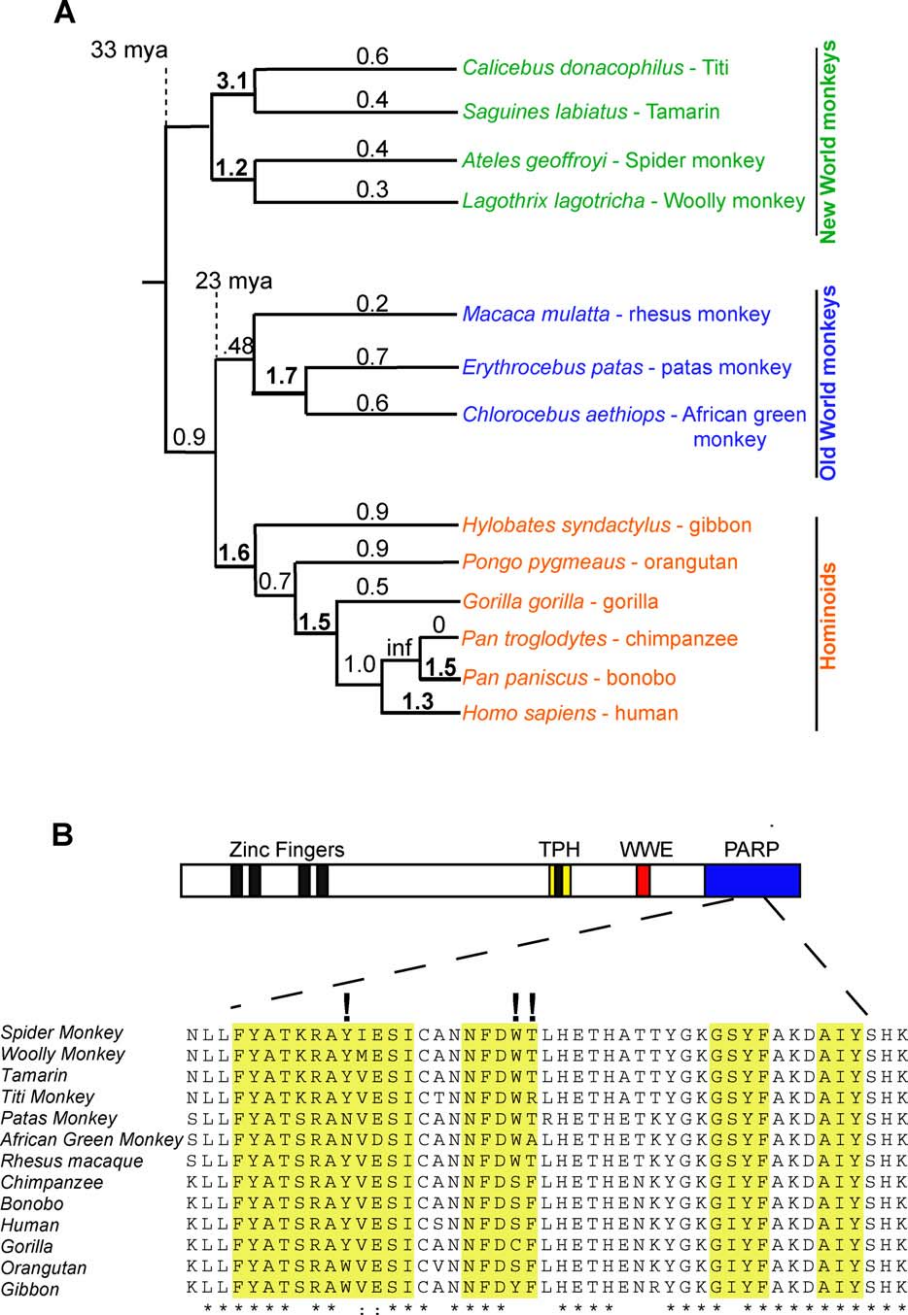
Positive Selection of *ZAP(L)* in Primates (A) Ancient signature of positive selection in primate ZAP. A sequence alignment of *ZAP(L)* from four New World monkeys, three Old World monkeys, and six hominoids was analyzed using the free-ratio model from PAML, which allows dN/dS to vary along each branch. The corresponding dN/dS values are shown for each branch, and bold numbers indicate those branches with dN/dS > 1. In the case of no observed synonymous changes (inf), a dN/dS ratio could not be calculated. (B) An alignment of a segment of the PARP domains from these primate species is shown. Identical and similar residues are indicated with asterisks (*) and colons (:), respectively. The 3 residues identified with high confidence as evolving under positive selection are indicated with exclamation marks and lie in close proximity to the NAD+ binding contact residues (indicated by yellow boxes).

To investigate whether the ZAP gene has evolved under positive selection during primate evolution, we sequenced the *ZAP(L)* coding sequence (2.7 kb) via RT-PCR from 13 primates representing 33 million years of evolution. Our analysis included 6 hominoids, 3 Old World monkeys (OWM), and 4 New World monkeys (NWM). The phylogeny constructed from the primate *ZAP* sequences was congruent with the generally accepted primate phylogeny confirming that the sequences are orthologous (Figure 2A). Using the free-ratio model in the PAML suite of programs [20], which allows an independent assignment of dN/dS ratios to each evolutionary branch, we found that several branches of the phylogeny show dN/dS > 1 (bold numbers in Figure 2A). Furthermore, when we compared the likelihood of *ZAP* evolution under codon models that prohibit (Nsites models M1, M7, or M8a) or permit positive selection (M2 and M8), we found that models permitting positive selection fit *ZAP* evolution significantly better than those that disallow it (*p* < 0.0003, Table 1). This indicates that *ZAP* has evolved under positive selection during primate evolution, suggesting it has a long history of actively participating in host–pathogen interactions.

**Table 1:**
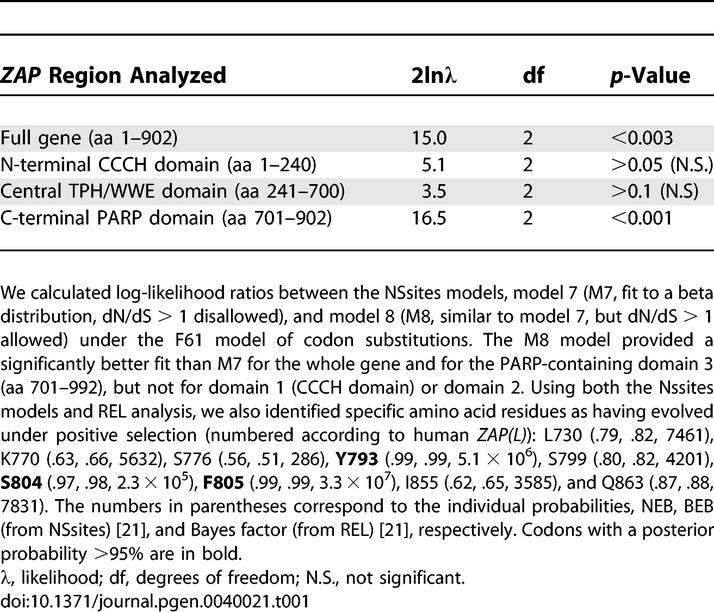
Likelihood Ratio Test Statistics for Models of Variable Selective Pressure Among Sites

To determine which domains were responsible for the signature of positive selection in ZAP, we performed three separate PAML analyses using the N-terminal CCCH domain (amino acids [aa] 1–240), the central domain containing the TPH and WWE motifs (aa 241–700), or the C-terminal PARP domain (aa 701–902). Significantly, our PAML analyses did not reveal any evidence for positive selection acting on the N-terminal CCCH domain. Indeed, we found a high degree of conservation of the CCCH domain throughout mammalian evolution (Figure S1), arguing that rapid alteration of viral RNA-binding has not had a significant impact on ZAP evolution. Sliding window dN/dS analyses based on different pairwise alignments of primate ZAP genes suggests that positive selection may have episodically acted on the central domain containing the TPH and WWE motifs (aa 241–700; Figure S2). However, since no codons show a recurrent signature of positive selection, these domains are not highlighted by the PAML analyses (Table 1).

Rather, we found a robust signature for positive selection in the PARP-like domain (aa 701–902; Table 1). We found that a model of episodic positive selection (Free Ratio) was significantly more likely than a model of constant positive selection (Model 0, one fixed dN/dS; *p* = .01, df = 21), suggesting that ZAP has been engaged in episodic conflicts with exogenous infectious agents. Three codons were identified as having evolved under recurrent positive selection in the PARP domain, using both PAML and REL analyses (Table 1) [20,21]. Surprisingly, the three residues for which we obtained high confidence of positive selection (Figure 2B) are found in close proximity to residues that are thought to mediate the contact residues for NAD+ binding (cd01439.2 from the CDD database [22] largely modeled from the crystal structure of the chicken PARP-1 catalytic domain [23,24]). This is highly unexpected because it would seem that these residues should be highly constrained as part of PARP or PARP-like function, for which NAD+ binding is obligatory. It is possible that these residues could be rapidly evolving because of ZAP(L) interactions with viral proteins that may involve the same protein domains that interact with NAD+. Thus, contrary to expectation, the only signature of recurrent positive selection we found in primate *ZAP* evolution is limited to a domain that is missing from the *rZAP* gene, which is the only version that has been previously tested in any antiviral assays. Since protein innovation has been primarily favored exclusively in the PARP domain, this would predict that ZAP(L) is the more evolutionarily relevant antiviral isoform. This prediction can be directly evaluated by testing whether the region under the most intense positive selection, the PARP-like domain, enhances the antiviral activity of ZAP.

### Human ZAP(L) Is a More Potent Viral Inhibitor than ZAP(S)

**Figure 3:**
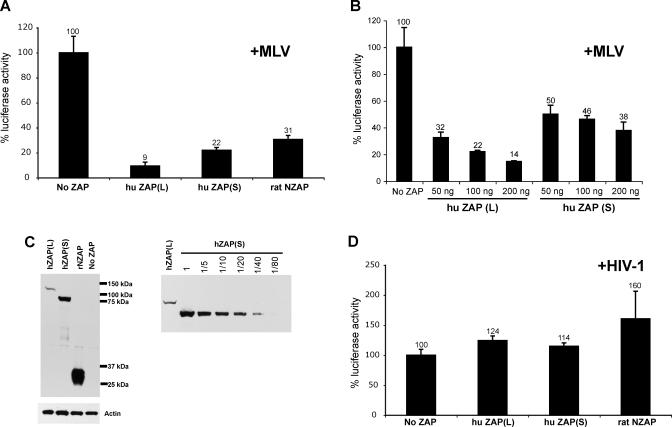
ZAP Viral Inhibition Is Specific to MLV but Not HIV, and the PARP Domain Enhances the Viral Restriction Co-transfections of ZAP isoforms with the LlucSN (MLV) or pwtLTR-luc1 (HIV) promoter constructs were used to assess the ability of the ZAP isoforms to restrict HIV- and MLV-driven luciferase. (A) ZAP inhibits MLV in cells transfected with equal amounts of the *ZAP* expression constructs. Equal amounts of each *ZAP* expression construct were co-transfected with LlucSN (MLV). All transfections were equalized for DNA amount with the addition of pcDNA. Lysates from transfected cells were collected after 24 h, and luciferase activity was measured. (B) Effect of increasing amounts of transfected human *ZAP(L)* or *ZAP(S)* DNA on MLV-LTR driven luciferase expression. Lysates from transfected cells were harvested at 48 h, and luciferase activity was measured. Results are shown as percent luciferase expression of the control transfection (no ZAP). The results from one representative experiment are shown, with error bars reflecting the variation between triplicate infections within one experiment. (C) Equivalent amounts of lysates from 293T cells transiently co-transfected with different amounts of *ZAP* expression constructs and the MLV LTR-*luciferase* construct were analyzed by Western blot analysis to determine the relative amount of protein. Samples were harvested 48 hours after transfection. Sizes of HA-tagged ZAP(L), ZAP(S), and rat NZAP are ∼115 kDa, ∼80 kDa, and ∼30 kDa, respectively. Actin was the loading control. To determine the relative amounts of ZAP(L) and ZAP(S), Western blot analysis was performed on a dilution series of lysates from 293T cells transfected with the *ZAP* constructs. (D) For ZAP and HIV co-transfections, pwtLTR-luc1 (HIV) was co-transfected with either no ZAP, ZAP(L), ZAP(S), or rat NZAP (a CMV-Tat construct was also transfected to improve expression levels of HIV). Transfections and luciferase assays were performed as in the MLV experiment.

The original identification of rat NZAP was based on its ability to inhibit MLV infection and MLV long terminal repeat (LTR)–based expression. We created HA-tagged human ZAP(L) and ZAP(S) expression constructs in parallel with the previously described rat NZAP clone [11] and then tested their restrictive capabilities by co-transfecting increasing amounts of ZAP plasmid with a MLV-based vector, in which the firefly luciferase reporter gene is inserted between the 5′ and 3′ LTRs of MLV. A similar MLV-based luciferase reporter construct was previously used to show that the 3′ LTR sequence of MLV is the target of the CCCH domain of rat NZAP [12]. In repeated luciferase assays (*n* = 6), we found that all ZAP expression plasmids significantly suppressed MLV LTR-driven luciferase expression (Figure 3A). Our experiments also show that ZAP(L) is a 2- to 3-fold stronger suppressor of MLV than ZAP(S) at the highest tested levels of ZAP (200 ng; Figure 3A–3B). In addition, Western analysis of lysates from the comparable transfections reveals that the ZAP(S) expression level is significantly higher than ZAP(L) (Figure 3C), despite equal amounts of transfected plasmid. This suggests a secondary control of ZAP(L) expression, which we have not explored further. However, these results do imply that the 2- to 3-fold higher inhibition of MLV expression seen with ZAP(L) is likely to be a significant underestimate. As a control, we also tested whether the ZAP isoforms were effective at inhibiting a similar construct that utilized the LTR from HIV to drive luciferase gene expression. We found that human ZAP(L), ZAP(S) and rat NZAP had no effect on HIV LTR-driven luciferase (Figure 3D), suggesting a strong specificity for retroviral inhibition, as was previously reported for rat NZAP [11]. These results demonstrate human *ZAP* has potent antiviral function, and that *ZAP(L)* is a more effective inhibitor than *ZAP(S).*

**Figure 4:**
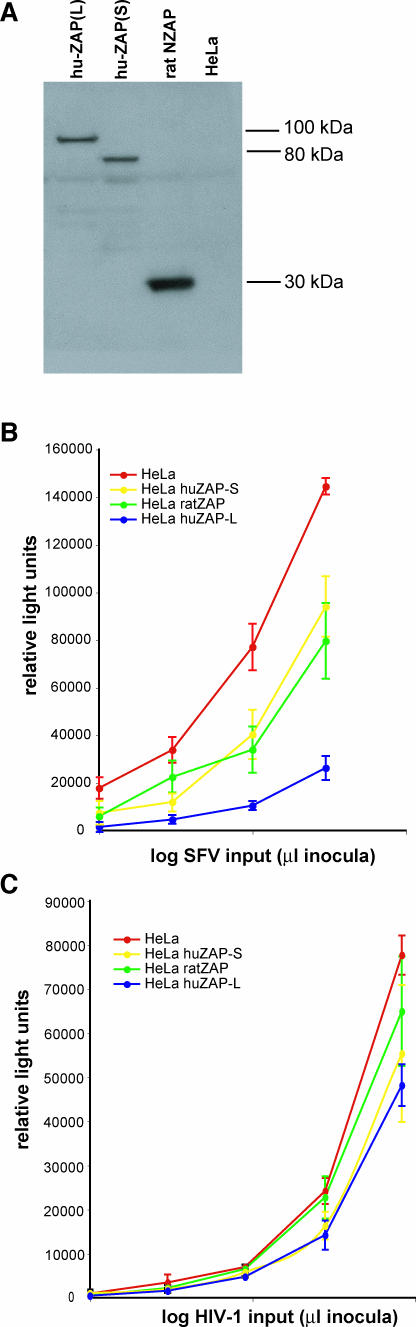
Human ZAP Restricts SFV Infection in Human Cells through the PARP Domain (A) Western analysis with anti-HA antibody shows expression of HA-tagged human and rat ZAP isoforms in stably expressing HeLa cell lines. Equal amounts of protein were loaded in each lane. (B) SFV infection was strongly inhibited (7-fold) in HeLa cells stably expressing ZAP(L), but only weakly restricted (2-fold) in HeLa cells stably expressing ZAP(S) or rat NZAP. Cells were challenged with four 3-fold serial dilutions of virus in quadruplicate and infectivity was determined by measuring SFV-driven β-galactosidase activity as relative light units using a luminescent substrate. The data are plotted as the means ± standard deviation on a log scale. (C) HIV infection was not attenuated in HeLA cells stably expressing the ZAP isoforms. HeLa cells stably expressing the ZAP isoforms were challenged by five 3-fold serial dilutions of virus, and infectivity was determined by measuring HIV-driven luciferase activity (relative light units). The data are plotted as the means ± standard deviations on a log scale.

Previous results have also demonstrated that rZAP can inhibit alphaviruses [14]. Therefore, we tested whether the two human ZAP isoforms were also capable of restricting an alphavirus, SFV, using a previously described DNA-based recombinant SFV vector system that expresses the β-gal gene [25]. We performed single-round infectivity assays in HeLa cells lines stably-expressing HA-tagged human ZAP(L), human ZAP(S), or rat NZAP. The expression level of ZAP(L) was about equal to that of ZAP(S), while rat NZAP appeared to be more highly expressed in our cell lines (Figure 4A). While ZAP(L), ZAP(S) and rat NZAP all demonstrate antiviral activity against SFV infection, ZAP(L) was significantly more potent than the other isoforms, with almost 10-fold inhibition of SFV compared to only 2-fold inhibition by either ZAP(S) or rat NZAP (Figure 4B). To assess whether the antiviral activity of human ZAP is a general antiviral effect (and to rule out the possibility that our observations are due simply to greater cell death in ZAP(L)-expressing cells), we performed single-round infectivity assays with HIV expressing luciferase in HeLa cells stably expressing the ZAP isoforms. We found that none of the isoforms of ZAP were capable of significantly inhibiting HIV infection, demonstrating that the antiviral activity of ZAP is virus-specific (Figure 4C). Intriguingly, the expression of human ZAP isoforms in baby hamster kidney (BHK) cells did not confer resistance to SFV (unpublished data), suggesting that the rapid evolution of ZAP has likely resulted in species-specific restriction and that ZAP restriction of alphaviruses is at least partially dependent on the context of other host proteins.

## Discussion

Our evolutionary analyses of primate *ZAP(L)* reveals that the C-terminal PARP-like domain encoded only by the *ZAP(L)* isoform is in fact the only domain that is under recurrent positive selection. Based on this insight from our evolutionary analysis, we tested the abilities of both human ZAP(L) and ZAP(S) isoforms to inhibit SFV and HIV infection in human cells. Our results clearly demonstrate that human ZAP can inhibit SFV, but not HIV, and that the presence of the PARP domain significantly enhances the ability of ZAP to suppress SFV infection. While previous work also implicated the rat ZAP as an inhibitor of the murine retrovirus, MLV, in rodent cells [11], we do not observe such an effect against MLV infection (unpublished data) at the level of ZAP expressed in our stable human cell lines, even though we do see an effect on SFV infection in the same cell lines. Therefore, we believe that the viral antagonist(s) that drove ZAP evolution in primates was likely a member of the *Togaviridae* family [14,15] rather than the *Retroviridae* family.

The rZAP CCCH domain can be very specific in its RNA-binding and antiviral activity [12]. Our experiments on MLV and HIV (Figure 3) suggest that the RNA-binding specificities of rodent and primate ZAP are not significantly different. Our analyses cannot rule out the possibility that some subtle alteration of RNA-binding may have occurred during the course of mammalian evolution. However, the absence of positive selection in the CCCH domain unambiguously rules out an evolutionary conflict scenario in which the restrictive ability of ZAP was shaped by repeated episodes of selection for dramatic alterations in RNA-binding activity.

It is not yet clear what role a PARP-like domain could play in ZAP function. PARP function has been primarily characterized in the nucleus where it is believed that the addition of ADP-ribose moieties to chromatin proteins results in their looser association with DNA, thereby allowing greater access to transcription and DNA repair machineries [19,26–28]. A similar mechanism of protein modification by ZAP's PARP domain could be imagined to disrupt binding by viral RNA-binding proteins (Figure S4). However, the ZAP PARP-like domain lacks what is believed to be an essential catalytic glutamic acid (position 988 in PARP-1) and therefore may not be catalytically active as a canonical PARP [29], although its NAD+ binding site is conserved.

While the mechanism by which the PARP-like domain mediates its antiviral effect is unknown, we can still address why the PARP-like domain has been evolving under positive selection. Using the model for antagonistic host–pathogen interactions, there are several possible scenarios for the adaptive evolution of the PARP-like domain in ZAP: either the PARP domain evolves towards recognizing the virus, or it evolves away from being recognized by the virus. In the first scenario, the PARP-like domain may increase ZAP's affinity for viral mRNA binding proteins (analogous to APOBEC3G associating with nucleocapsid [30]), in which case the PARP-like domain is under selective pressure to *increase binding* to viral proteins. The alternate possibility is that viruses may encode specific antagonists to bind ZAP's PARP domain and sequester or degrade ZAP (analogous to Vif degradation of APOBEC3G [31]), in which case the selective pressure on ZAP PARP would be to *decrease binding* with viral antagonists. We favor the latter possibility because the residues that show a recurrent signature of positive selection also fall proximal to the NAD+ binding sites, which may be essential for any PARP-like function. Given the recent re-emergence of alphavirus epidemics in Africa and Asia and the reports of alphavirus disease in Europe [32,33], our discovery that human ZAP encodes a potent anti-alphaviral activity that depends on the PARP domain may guide future strategies for therapeutic drug design to treat alphavirus-related disease.

## Materials and Methods

### RT-PCR and sequencing.

Human, chimp, and rhesus *ZAP* sequences were obtained from the respective genome projects. We amplified *ZAP* genes from additional primates (Figure 2A) by RT- PCR using RNA isolated from individual cell lines (obtained from Coriell and E. Eichler [gibbon]). RT-PCR was done in two overlapping fragments using the Invitrogen One-Step RT-PCR with Platinum Taq kit (Invitrogen). To amplify the 5′ half of the gene, we used the following primers to generate a 1.4 kb fragment: Forward-5′ ATGGCGGACCCGGAGGTG, Reverse-5′ CTCGGGAAGCAGGTCCAGCATCC. The 3′ half of the gene was amplified as a 1.4 kb fragment with the following primers: Forward-5′ AATGCTGATGGAGTGGCCACAG, Reverse-5′ GACAACTAACTAATCACGCAGGCTTTGTC. To demonstrate the existence of a PARP-containing rat *ZAP (L),* the following primers were used to generate a fragment spanning exons 7–13 of *ZAP*: Forward-5′ TCTGACTCCTACCCCATCCGA, Reverse-5′ GCAACCTTTCTCTTTCTCTGATTCCAC. All sequencing was done using ABI BigDye version 3.0.

### Nucleotide sequence accession numbers.

Sequences obtained in this study were deposited in Genbank and assigned accession numbers EF494425–EF494434.

### Expression panels.

To detect *ZAP* expression in different human tissues, we used human cDNA panels in which 5 ng of first-strand cDNA from various tissues were preloaded (Primgen) and performed PCR using primers specific to either the ZAP(S) or ZAP(L) isoform.

### Evolutionary analyses.

We used Clustal_X [34] to generate a multiple alignment for ZAP from all primate species sequenced. Maximum likelihood analysis was performed with codeml in the PAML 3.14.1 software package [35]. To detect selection, multiple alignments were fitted to the NSsites models that disallow positive selection (M1, M7, M8a) or to models that permit positive selection (M2, M8, M8b, respectively) assuming the f61 model of codon frequencies. Simulations were run with multiple starting values for dN/dS. Likelihood ratio tests were performed to assess whether permitting codons to evolve under positive selection gives a significantly better fit to the data. To identify specific codons that are evolving under recurrent positive selection, we used the NSsites model from codeml in the PAML 3.14.1 software. To confirm the sites identified by the codeml approach, we also implemented the random effects method (REL) from the online DataMonkey package, with a Bayes significance factor of 50 as the cutoff [21]. The REL approach differs from NSsites in that it allows the synonymous substitution rate to vary among codons. Our pairwise sliding window analyses were performed using the K-estimator program [36], with a 300 bp window and a 50 bp slide.

### Expression constructs.

N-terminal hemagglutinin (HA)-tagged human ZAP(L) and ZAP(S) were cloned by reverse transcription (RT)-PCR from RNA isolated from 293T cells using the HA-specific 5′ primer, 5′-CAGGCGAATTCGCCACCATGTATCCATACGATGTTCCAGATTACGCTGCGGACCCGGAGGTGTGC-3′, and isoform-specific 3′ primers as follows: HA-ZAP(L)-5′ TTCAGGATATCCTAACTAATCACGCAGGC-3′ and HA-ZAP(S)-5′-TTCAGGATATCCTATCTCTTCATCTGCTGCAC-3′. The rat N-terminal HA-tagged NZAP was cloned by RT-PCR from RNA isolated from Rat2 fibroblasts using the HA-specific 5′ primer, 5′-CAGGCGAATTCGCCACCATGTATCCATACGATGTTCCAGATTACGCTGCAGATCCCGGGGTA-3′ and the 3′ primer, 5′-TTCAGGATATCTCAGTGAAGGAAGCGGTCTCT-3′. Each gene was transferred into the same mammalian expression vector (pcDNA4; Invitrogen) which drives gene expression under the cytomegalovirus IE94 (CMV) promoter. All constructs were confirmed by sequencing.

### Cell culture.

HeLa cells were cultured in Dulbecco's modified Eagle medium (DMEM) with 10% fetal bovine serum and were maintained at 37 °C in 5% CO_2_. To create stably expressing cell lines, the ZAP constructs were individually transfected into HeLa cells using Fugene6 Reagent (Roche) and stably expressing lines were selected and maintained with Zeocin (0.1 mg/ml). The expression levels for clonally derived lines were checked by Western analysis using an HA-specific antibody (HA.11; Covance) and the highest expressing lines were chosen for the infectivity assays.

### Transient cotransfection, luciferase assay, and Western blot analysis.

Transient co-transfections were performed using the Mirius TransIT-LTR Transfection reagent according to the manufacturer's recommendations. All cells were transfected with a total of 300 ng DNA comprising 100 ng LlucSN plus empty pcDNA4 vector (No ZAP), HA- ZAP expression plasmid (HA-hZAPL, HA-hZAPS, or HA-rNZAP), as indicated, and the total DNA transfected was equalized with empty pcDNA4 vector. After 48 h, the cells from triplicate tranfections were lysed using 150 μl of Cell Culture Lysis reagent (Promega). To quantify the luciferase activity, 20 μl of lysate was analyzed with a Luciferase Assay kit (Promega) and luciferase activity (light intensity) was measured with a luminometer.

For Western blot analysis, 293T cells were plated and transfected as described above using different amounts of transfected HA-huZAP(L) or HA-huZAP(S), with the total amount of transfected DNA equalized using empty pCDNA4. After 48 h, the lysates from these cells were harvested with NP-40-doc buffer (20 mM Tris [pH 8.0], 120 mM NaCl, 1 mM EDTA, 1% NP-40, 0.2% Na-deoxycholate, and protease inhibitors [Roche complete cocktail tablets]), and incubated on ice for 5 min and then frozen at −20 °C. Lysates were resuspended in SDS loading buffer, boiled for 5 min, and then loaded and run on a 12% NuPAGE Novex Bis-Tris Gel and transferred to nitrocellulose membrane (Pierce). For HA-tagged ZAP protein detection, membranes were probed with a HA-specific antibody (HA.11; Covance) at a 1:2,000 dilution, followed by horseradish peroxidase (HRP)-conjugated goat anti-mouse secondary antibody (Amersham Biosciences) at a 1:3,000 dilution. For Actin protein detection, a rabbit anti-Actin Ab (Sigma) was used at 1:10,000 dilution, followed by HRP-conjugated donkey anti-rabbit secondary antibody (Amersham Biosciences) at 1:3,000. Detection was performed with the ECL Plus Reagent (Amersham Biosciences).

### Virus stocks.

SFV virus was made using the DNA-based Semliki Forest Virus vectors (pSMARTlacZ and pSCAHelper), which were a kind gift from Dr. Rod Bremner [25]. To make HIV virus stock we used HIV-luc2Δenv pseudotyped with VSV-G [37]. To make the virus stocks, the constructs were transiently transfected into 293T cells with FuGene6 Reagent (Roche). Virus released into the cell culture supernatant by 48 h was harvested, clarified by centrifugation, and stored at −80 °C.

### Single-cycle infectivity assays.

SFV infections were performed as previously described [25]. Briefly, cells were plated in 96 well plates at 2 × 10^4^ cells per well the day before infection. Virus was thawed and treated with chymotrypsin to generate active virus and HeLa cell lines (normal and stably expressing ZAP isoforms) were challenged with 3-fold serial dilutions of active virus. After 24 h, the cells were lysed and the β-galactosidase activity was measured using Galacton (Tropix) as a substrate with 1-s measurements using a luminometer (Thermo Fluoroskin Ascent, Thermo).

For the HIV infections, cells (normal or stably expressing ZAP isoforms) were plated in 96-well plates at 2 × 10^4^ cells per well and after 24 h were challenged for 48 h with serial dilutions of HIV virus. After 48 h, the cells were lysed using the Cell Culture Lysis reagent (Promega). To quantify the luciferase activity, the lysate was analyzed with a Luciferase Assay kit (Promega) and luciferase activity (light intensity) was measured with a luminometer.

## Supporting Information

Figure S1 Sliding Window dN/dS Analysis of Primate *ZAP* Genes Sliding window (300 bp window, 50 bp slide) analyses of dN and dS were performed, and 3 representative pairs of primate ZAP genes are shown: (A) HOM-OWM (human versus patas monkey), (B) HOM-NWM (human versus woolly monkey), and (C) OWM-NWM (rhesus versus spider monkey). For each pairwise comparison, dN/dS (thick line) and dS (thin line) are plotted against the length of the protein with the schematic of the protein shown at the bottom. A dotted line represents where dN/dS = 1, consistent with the neutral expectation.
(270 KB PDF)

Figure S2 Multiple Sequence Alignment of Orthologous ZAP N-Terminal Domains An alignment of the first 230 amino acids of ZAP, corresponding to the CCCH zinc finger motifs, from 14 primates and six non-primate mammals. Residues are highlighted in black or gray to indicate complete conservation or high conservation, respectively. The putative NLS is indicated between residues 69 and 76 and the four CCCH fingers are shown below the alignment.
(483 KB PDF)

Figure S3 ZAP Paralogs in Vertebrates (A) There are at least four other proteins that share common structural features with ZAP. ZRP2, PARP12, and ZAP are located on 7q34; TiPARP is on 3q25; and PARP11 is on 12p13. Only ZAP is evolving under positive selection (red arrow).
(B) ZRP2 (NP_542391) is a predicted 300 amino acid protein that contains the four CCCH type zinc fingers found in ZAP. PARP12 and ZAP share the CCCH fingers, the TPH (including 1 CCCH finger), WWE, and PARP-like domain. PARP11 and TiPARP lack the CCCH domain but both contain the WWE and PARP-like domain.
(221 KB PDF)

Figure S4 A Hypothetical Model for How ZAP PARP-Like Activity Might Enhance its Antiviral Function (A) In the nucleus, PARP adds poly(ADP-ribose) (PAR) moeities to chromatin proteins, thus loosening their association with DNA and opening the chromatin structure, which allows greater access to transcription and DNA repair machineries. This opening of the chromatin structure is reversed by PARG, a glycohydrolase that mediates the breakdown of PAR.
(B) Our model proposes that ZAP restricts viral replication by adding PAR moieties to proteins associated with viral mRNAs in the cytoplasm, thus weakening their interaction and exposing the mRNA to exosomal degradation. Although PAR moeities are shown here, even addition of Mono (ADP-ribose) could mediate the same effects. It is presently unclear whether ZAP's PARP domain possesses such catalytic activity, since it is missing one of the critical catalytic residues previously thought to be essential for PARP activity.
(708 KB PDF)

## Abbreviations

APOBEC3G: apolipoprotein B editing complex-like protein 3G
dN: number of non-synonymous substitutions per site
dS: number of synonymous substitutions per site
PARP: poly(ADP-ribose) polymerase
TRIM5α: tripartite motif protein 5 alpha
ZAP: zinc-finger antiviral protein
