# Supplementary figures and images for: Positive Selection and Increased Antiviral Activity Associated with the PARP-Containing Isoform of Human Zinc-Finger Antiviral Protein
Source: PLoS Genet. 2008 Jan 25;4(1):e21. doi: 10.1371/journal.pgen.0040021 (PMC2213710; doi:10.1371/journal.pgen.0040021)

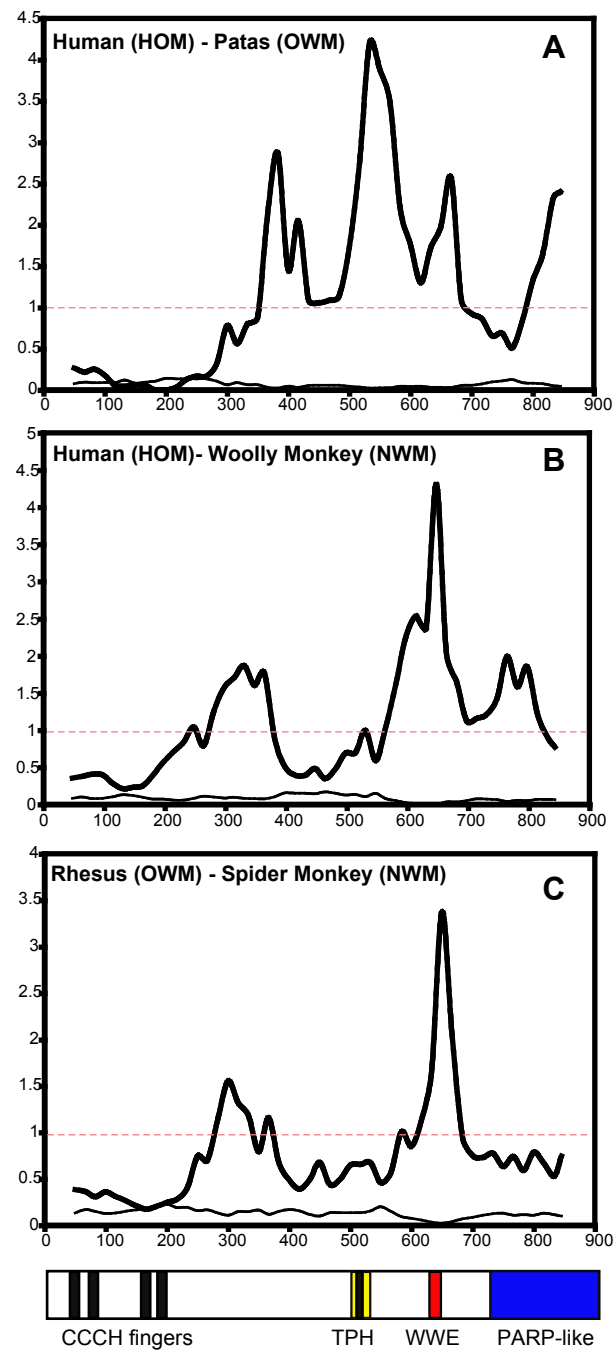

**Kerns et al Supplementary Figure 1**

Supplement: Figure S1 — Sliding window (300 bp window, 50 bp slide) analyses of dN and dS were performed, and 3 representative pairs of primate ZAP genes are shown: (A) HOM-OWM (human versus patas monkey), (B) HOM-NWM (human versus woolly monkey), and (C) OWM-NWM (rhesus versus spider monkey). For each pairwise comparison, dN/dS (thick line) and dS (thin line) are plotted against the length of the protein with the schematic of the protein shown at the bottom. A dotted line represents where dN/dS = 1, consistent with the neutral expectation. (270 KB PDF) [file pgen.0040021.sg001.pdf]

**A**

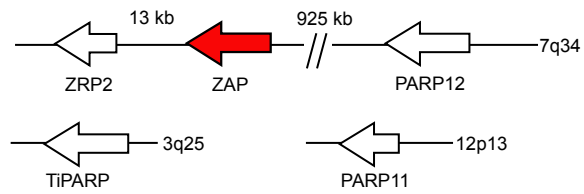

**B**

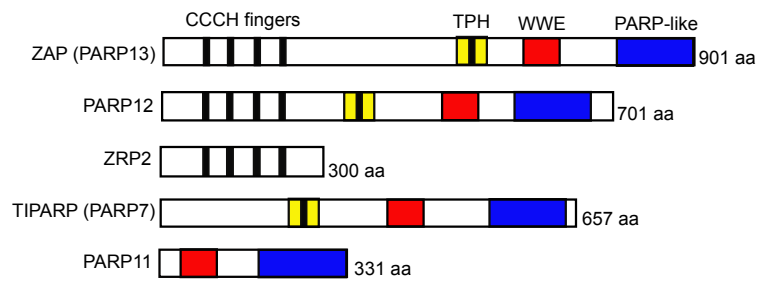

**Kerns et al Supplementary Figure 3**

Supplement: Figure S3 — (A) There are at least four other proteins that share common structural features with ZAP. ZRP2, PARP12, and ZAP are located on 7q34; TiPARP is on 3q25; and PARP11 is on 12p13. Only ZAP is evolving under positive selection (red arrow). (B) ZRP2 (NP_542391) is a predicted 300 amino acid protein that contains the four CCCH type zinc fingers found in ZAP. PARP12 and ZAP share the CCCH fingers, the TPH (including 1 CCCH finger), WWE, and PARP-like domain. PARP11 and TiPARP lack the CCCH domain but both contain the WWE and PARP-like domain. (221 KB PDF) [file pgen.0040021.sg003.pdf]

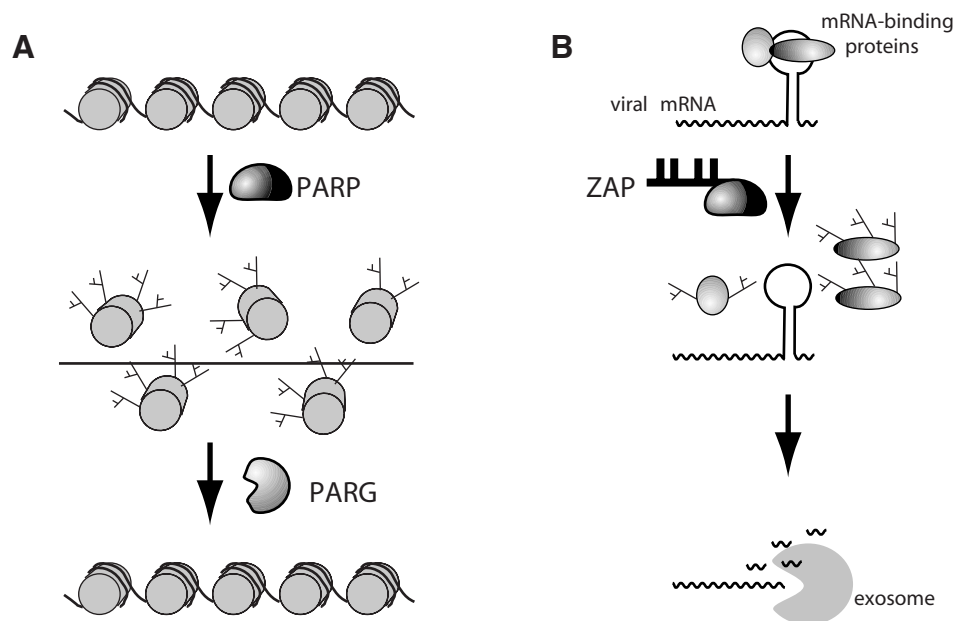

**Supplementary Figure 4**

Supplement: Figure S4 — (A) In the nucleus, PARP adds poly(ADP-ribose) (PAR) moeities to chromatin proteins, thus loosening their association with DNA and opening the chromatin structure, which allows greater access to transcription and DNA repair machineries. This opening of the chromatin structure is reversed by PARG, a glycohydrolase that mediates the breakdown of PAR. (B) Our model proposes that ZAP restricts viral replication by adding PAR moieties to proteins associated with viral mRNAs in the cytoplasm, thus weakening their interaction and exposing the mRNA to exosomal degradation. Although PAR moeities are shown here, even addition of Mono (ADP-ribose) could mediate the same effects. It is presently unclear whether ZAP's PARP domain possesses such catalytic activity, since it is missing one of the critical catalytic residues previously thought to be essential for PARP activity. (708 KB PDF) [file pgen.0040021.sg004.pdf]
