# Supplementary material for: Positive Selection and Increased Antiviral Activity Associated with the PARP-Containing Isoform of Human Zinc-Finger Antiviral Protein
Source: PLoS Genet. 2008 Jan 25;4(1):e21. doi: 10.1371/journal.pgen.0040021 (PMC2213710; doi:10.1371/journal.pgen.0040021)
Supplement: Figure S2 — An alignment of the first 230 amino acids of ZAP, corresponding to the CCCH zinc finger motifs, from 14 primates and six non-primate mammals. Residues are highlighted in black or gray to indicate complete conservation or high conservation, respectively. The putative NLS is indicated between residues 69 and 76 and the four CCCH fingers are shown below the alignment. (483 KB PDF) [file pgen.0040021.sg002.pdf]

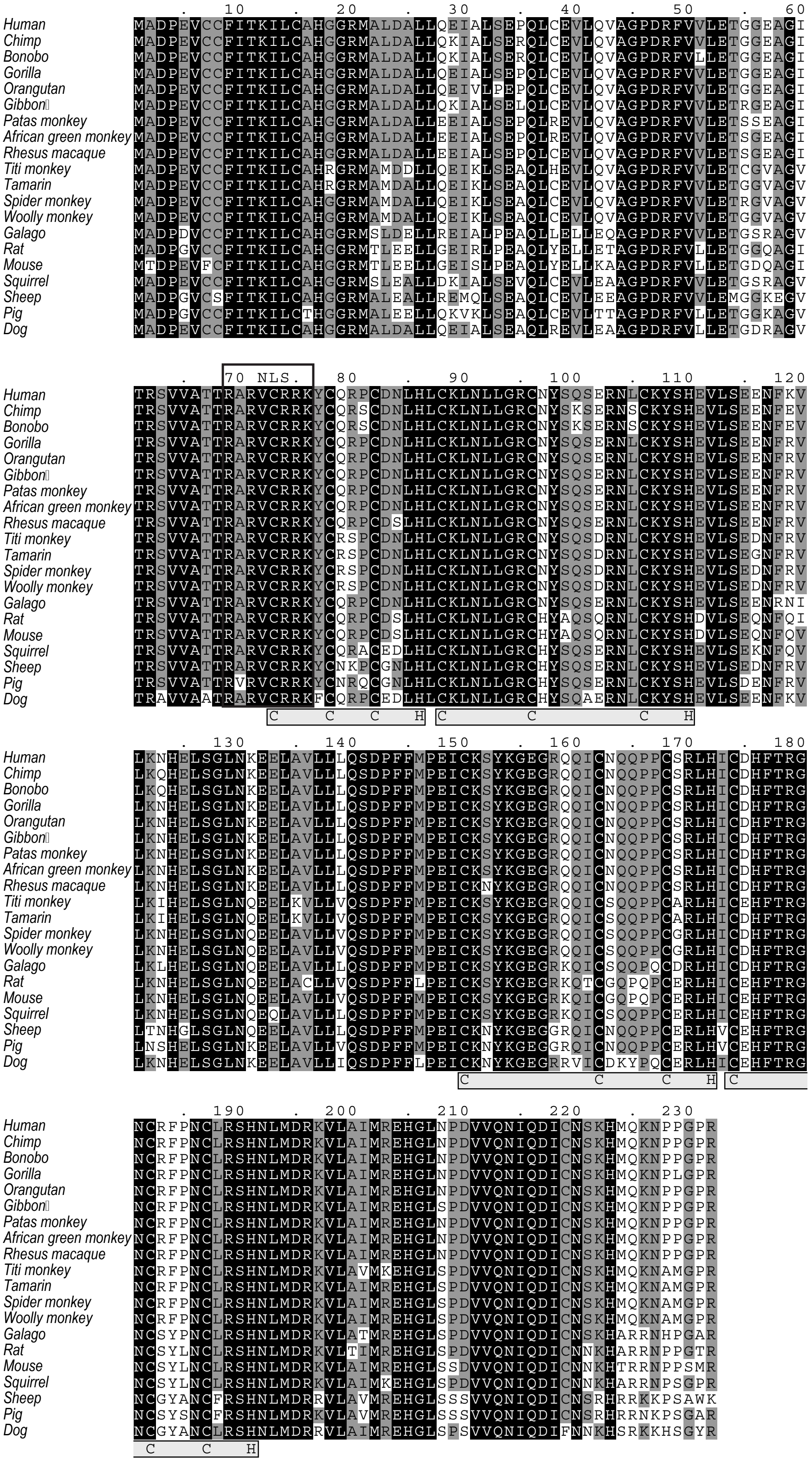


**Kerns et al Supplementary Figure 2**
